# Author Correction: A self-amplifying RNA vaccine against COVID-19 with long-term room-temperature stability

**DOI:** 10.1038/s41541-022-00578-7

**Published:** 2022-11-23

**Authors:** Emily A. Voigt, Alana Gerhardt, Derek Hanson, Madeleine F. Jennewein, Peter Battisti, Sierra Reed, Jasneet Singh, Raodoh Mohamath, Julie Bakken, Samuel Beaver, Christopher Press, Patrick Soon-Shiong, Christopher J. Paddon, Christopher B. Fox, Corey Casper

**Affiliations:** 1 RNA Vaccines, Access to Advanced Health Institute (AAHI), Seattle, WA 98102 USA; 2 Product Development, Access to Advanced Health Institute (AAHI), Seattle, WA 98102 USA; 3 Formulation Sciences, Access to Advanced Health Institute (AAHI), Seattle, WA 98102 USA; 4 ImmunityBio, Inc, Culver City, CA 90232 USA; 5 Amyris, Inc, Emeryville, CA 94608 USA; 6 Department of Global Health, University of Washington, Seattle, WA 98195 USA; 7 Department of Medicine, University of Washington, Seattle, WA 98195 USA; 8 Vaccine and Infectious Disease Division, Fred Hutchinson Cancer Center, Seattle, WA 98109 USA

**Keywords:** RNA vaccines, RNA vaccines

Correction to: *npj Vaccines* 10.1038/s41541-022-00549-y, published online 02 November 2022

In the original version of this Article, the α symbols were missing for TNFα in Figure 5f and 5g.

Original Fig. 5
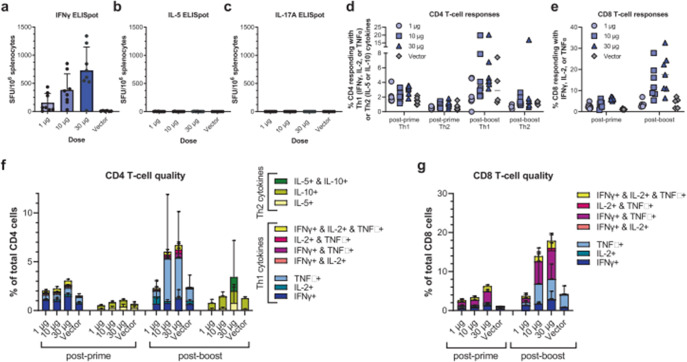


Revised Fig. 5
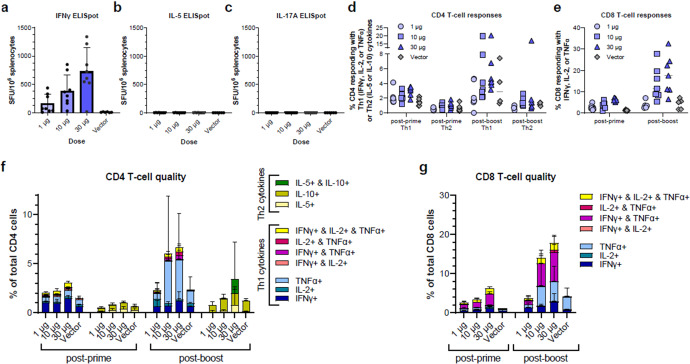


The figure in the HTML and PDF versions of the Article has now been updated.

